# TSH secreting adenoma: a rare cause of severe headache

**DOI:** 10.11604/pamj.2016.23.2.8451

**Published:** 2016-01-08

**Authors:** Serdar Olt, Mehmet Şirik

**Affiliations:** 1 Adıyaman University Medical Faculty Department of Internal Medicine, Adıyaman, Turkey; 2 Adıyaman University Medical Faculty Department of Radiology, Adıyaman, Turkey

**Keywords:** TSH secreting adenoma, severe headache, hyperthyroïdism

## Image in medicine

31 years old male patient admitted to the emergency department because of recurrent severe headaches which continued for two years. The patient has been consulted our clinic of internal medicine because of thyroid function abnormalities. Laboratory investigations revealed elevated serum TSH of 8,6 mU/L (normal range (N); 0.34-5,6), free T4 (fT4) of 1,73 (N;0,61-1,12ng/dl), and free T3(fT3) of 5,48 (N; 2,5-3,9pg/mL). Other laboratory parameters were normal. Physical examination revealed stage 3 goiter. The other system examinations were normal. We have learned that two years ago brain computed tomography scan was performed due to headache and result was reported as normal. Considering secondary hyper thyroidism pituitary MR was performed. Pituitary MR revealed a 13x18 mm macro adenoma (Figure). The patient underwent transphenoidal surgery. Final diagnosis was TSH secreting adenoma after pathological examination. After surgery headache and hyperthyroidism were recovered.

**Figure 1:**
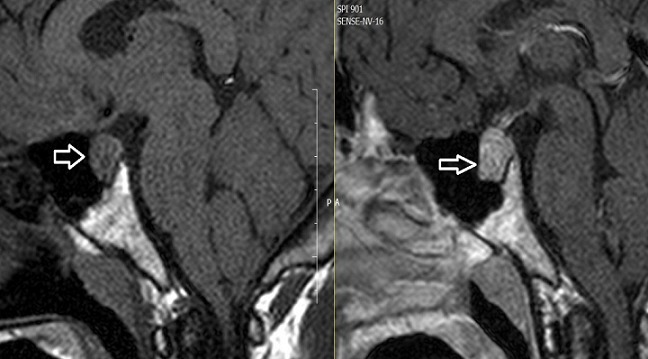
Non-contrast and contrast enhanced T1A image of pituitary macroadenoma

